# Microwave-assisted synthesis of titania–amorphous carbon nanotubes/amorphous nitrogen-doped carbon nanotubes nanohybrids for photocatalytic degradation of textile wastewater

**DOI:** 10.1039/d0ra08191d

**Published:** 2021-02-08

**Authors:** Sithembela A. Zikalala, Mandla B. Chabalala, Nozipho N. Gumbi, Neil J. Coville, Bhekie B. Mamba, Bridget K. Mutuma, Edward N. Nxumalo

**Affiliations:** Institute for Nanotechnology and Water Sustainability, College of Science, Engineering and Technology, University of South Africa Florida 1709 Johannesburg South Africa nxumaen@unisa.ac.za +27 11 670 9498; DSI-NRF Centre of Excellence in Strong Materials and Molecular Sciences Institute, School of Chemistry, University of the Witwatersrand Johannesburg 2050 South Africa; State Key Laboratory of Separation Membranes and Membrane Processes, National Centre for International Joint Research on Membrane Science and Technology Tianjin 300387 People's Republic of China

## Abstract

The synthesis of TiO_2_ nanohybrids fabricated using amorphous carbon nanotubes (aCNTs) and amorphous nitrogen doped carbon nanotubes (aNCNTs) *via* a microwave-assisted hydrothermal method is reported. The photocatalytic removal of Reactive Red 120 (RR 120) and organics from industrial textile wastewater using these nanohybrids is discussed. The synthesis process was shown to promote the removal of nano graphitic flakes from the outer walls of the aNCNTs and aCNTs and subsequent incorporation of these carbonaceous materials into TiO_2_ nanocrystals as such enabling a stronger interaction between the TiO_2_ and the carbonaceous material. This enabled the production of a surface plasmon resonance on the TiO_2_ and NTiO_2_ nanocrystals. The carbon residue was confirmed to be aCNTs and aNCNTs by TGA and DTA analyses. XPS analysis for the TiO_2_–aNCNT nanohybrids confirmed the C and N doping of TiO_2_ due to the amorphous residues from the aNCNTs. In addition, XPS and FTIR spectroscopic analysis confirmed the presence of surface oxygen-based groups. TEM micrograph analysis showed that aCNTs and aNCNTs promote the formation of monodispersed and small TiO_2_ particles; all below 7.4 nm. The NTiO_2_–aNCNT nanohybrids have the lowest energy band gap at 2.97 eV and the lowest PL intensity. The TiO_2_–aNCNT nanohybrids had superior adsorptive (98.2%) and photocatalytic (99%) removal for 20 ppm RR 120 dye solution at *k*_1app_ 3.44 × 10^−2^ min^−1^. Lastly, all the nanohybrids demonstrate the formation of visible-light absorbing intermediates from VAT-dyed textile wastewater. The work demonstrates the possibility of the use of these nanohybrids to derive new products through photocatalytic nanohybrids.

## Abstract 2

The synthesis of TiO_2_ nanohybrids fabricated using amorphous carbon nanotubes (aCNTs) and amorphous nitrogen doped carbon nanotubes (aNCNTs) *via* a microwave-assisted hydrothermal method is reported.

## Graphical abstract

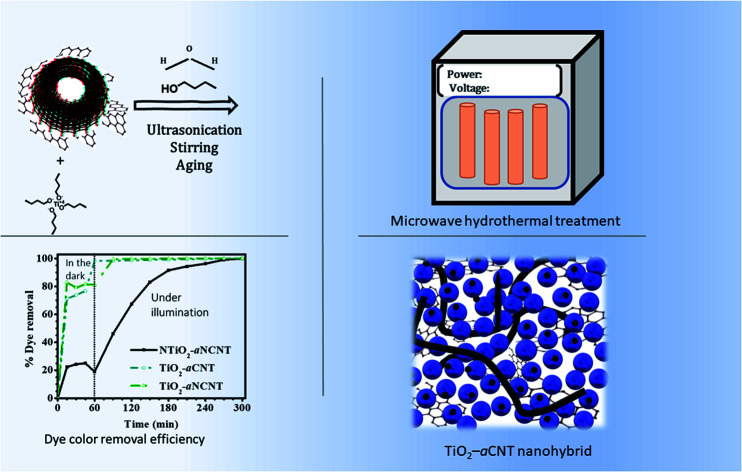

## 1. Introduction

Carbonaceous materials have received much attention due to their potential in enhancing the photocatalytic properties of TiO_2_ and its doped forms. Among many carbonaceous materials, carbon nanotubes (CNTs), carbon nanofibers (CNFs) and graphene are most widely used as support materials for TiO_2_ since they enhance the photocatalytic activity of TiO_2_ and its doped forms in several ways.^1^ First, their electron-rich surface enhances the absorption of light through photosensitization.^2^ Second, the high electrical conductivity of these carbonaceous supports decreases the electron–hole recombination (also known as charge recombination) in TiO_2_ hence making both photo-generated electrons (e^−^) and positive holes (h^+^) available to take part in the photocatalytic processes. In addition, these carbonaceous materials narrow the energy band gap (*E*_g_) in TiO_2_ thus enabling the absorption of visible light by TiO_2_.^3,4^

CNTs are cylindrical, tubular graphitic sheets that range from a single ring (single walled CNTs) to multiple concentric rings (multiwalled CNTs).^5^ The cylindrical morphology imputes CNTs with a high length to diameter ratio; a characteristic that makes them to have a high surface area.^6^ CNTs are renowned for their high tensile strength and high electrical conductivity; properties that are primarily due to the sp^2^ hybridization of the lattice carbons resulting in σ–σ, C–C bonds and π–π bonding resulting from the lone pair electrons on the carbons within the graphitic layer.^7^ Defects on these graphitic walls are characterized by sp^3^ hybridized carbons and as such decrease mechanical strength of CNTs. The introduction of heteroatoms into the graphitic lattice induces strain on the walls due to unequal bond strengths and lengths between the –C–C– and the carbon–heteroatom bond.^8^ The effect of heteroatom doping on the electrical conductivity, however, is dependent on electronegativity of the non-carbon atom relative to carbon. Electronegative and electron rich elements typically increase electrical conductivity.^9^

The quality of carbon nanotubes is also affected by the synthesis reaction environment in that low quality, thick-walled and less crystalline carbon nanotubes are produced when the synthesis times are longer temperatures, and carbon source concentrations are higher.^10,11^ Previous research has shown that chemical vapor deposition synthesis of CNTs at times longer than 1 h, high precursor and carrier gas flow rates and temperatures higher than 800 °C, results in the formation of CNTs with wide inner and outer diameters due to thick, but weak walls.^10^ While the inner layers of the walls may be graphitic, the outer layers generally consist of weakly held nano graphitic sheets and some amorphous residue.^12,13^ This makes the synthesis of such low-quality carbon nanotubes cheaper and as such appealing for the modification of semiconductors for catalytic applications. These types of CNTs are called amorphous carbon nanotubes (aCNTs).^13,14^

aCNTs have unique properties that make them find a different niche in several applications that crystalline CNTs do not occupy. The discontinuity of the walls imputes aCNTs with electrical and thermal properties that enable them to be good absorbers of electromagnetic radiation, have a large surface area, and an energy band gap (*E*_g_) that is inversely proportional to the diameter. aCNTs also exhibit relatively the same electronic properties regardless of the chirality. This suggests aCNTs are suited for the attachment of nanoparticles; a feature attributed to the high density of structural defects on the outer surface of aCNTs.^15^ Additionally aCNTs are resistant to oxidation in air at temperatures below 300 °C.^16^ These properties have made aCNTs to be widely used in such processes as adsorption,^17^ lithium-ion storage cells,^18^ space technology.^19^ The unique electrical properties of aCNTs also make them suitable for use in photocatalytic degradation experiments.^16,20^

As such, aCNTs and the amorphous residue, have been explored in the modification of semiconductor photocatalysts and found to demonstrate exceptional photocatalytic performances.^12,21,22^ For example, the degradation of methylene blue (MB) using amorphous carbon (aC) coated TiO_2_ was found to follow the first order kinetics at rate constant of 16.4 h^−1^ as opposed to that of bare TiO_2_ (4.14 h^−1^).^23^ The synergistic effect of doping TiO_2_ with N and hybridizing it with amorphous C increased visible light photosensitivity and extended the band edge of the nanohybrid up to 600 nm (which corresponds to *E*_g_ ∼ 2.36 eV).^21^ aCNTs have also been reported in the fabrication of Ag–aCNT and MnO–aCNT nanohybrids formed by supporting Ag and MnO nanoparticle onto aCNTs.^24,25^ The Ag–aCNT nanohybrids had a lower energy *E*_g_ than the Ag.^24^ In the latter case, aCNTs were shown to photosensitize MnO in visible light and extend its absorption edge into the visible range.^25^ A mechanically weak form of nitrogen doped carbon was used to form a composite with AgPO_4_ and found to enhance the charge separation and hence the photocatalytic performance of the nanocomposite.^26^

Due to the bulk of semiconductor–crystalline CNT nanohybrids, concerns on these nanohybrids compromising the filtration efficiency of polymer membranes through the formation of macropores at the polymer/nanoparticle interface have been raised.^27^ This has led to the carbonaceous material–crystalline CNT nanohybrids having seen less transition into membrane technology.^27^ As such, more uniform and smaller carbonaceous material–crystalline CNT material nanohybrids are desirable to impute photocatalytic properties onto polymers while ensuring the sustainable use of semiconductor-based photocatalysts without compromising the filtration capabilities. aCNTs are promising candidates for the tuning of the optical properties of semiconductor photocatalysts with a possibility of maintaining a strong interaction between the TiO_2_ and the carbonaceous material.

The aCNTs and aNCNTs used in this work were tailored to have loosely held graphitic nano flakes to investigate the contribution of the amorphous nature of aCNTs on the optical, chemical and mechanical properties of TiO_2_ and NTiO_2_. The nanotubes used in this study are characterized by thick walls in the range 40–100 nm, wider inner and outer diameters in the range 100–300 nm and bolus capping or open ends (Fig. 3(g)). Furthermore, addition of surface groups and doping with non-carbon elements into the CNT layers, makes the CNTs more amorphous and more prone to breaking down to shorter fragments and under extreme mechanical strain and extreme oxidizing environments to a mix of graphitic and amorphous residue.^28^ As such, it is expected that aNCNT would be fragmented to smaller graphitic and amorphous carbon residues which would be incorporated into or onto the NTiO_2_ nanoparticles.

In this study, an investigation of the influence of aCNT and aNCNTs on the properties of TiO_2_ and NTiO_2_ was conducted to study the effects of the amorphous nature of a CNT wall on its interaction with TiO_2_. *In situ* polymeric condensation of TiO_2_ and NTiO_2_ in an aCNT/aNCNT suspension was carried out using the microwave assisted hydrothermal method. These resultant photocatalytic nanohybrids were then evaluated for the degradation of RR 120 as a model dye. Finally, the nanohybrids were evaluated for the removal of color and organics in industrial textile effluent.

## 2. Materials and methods

### 2.1. Materials

Titanium butoxide (TiOBut), propanol, butanol, ethanol (Et·OH), acetylacetonate, FeCl·6H_2_O, Co(NO_3_)·6H_2_O and NH_4_OH, CaCO_3_ were all supplied by Sigma Aldrich South Africa. C_2_H_2_ and N_2_ gases supplied by Afrox South Africa, a tube furnace with an effective heating length of 80 cm equipped with a thermostat and a 1 m quartz tube were employed for the synthesis of aCNTs and aNCNTs. The synthesis of the TiO_2_–aCNT, TiO_2_–aNCNT and NTiO_2_–aNCNT nanohybrids was carried out in an MDS-6G synthesis/digestion microwave (Sineo-China) equipped with a reactor system that consists of Teflon-lined reactor vessels each covered with a SiC composite. Each vessel is held in a blast safety casing to enable pressure build up during synthesis.

### 2.2. Synthesis of aCNTs and aNCNTs

The Fe–Co/CaCO_3_ bimetallic catalyst was synthesized using the wet impregnation technique adapted from previous procedures using FeCl·6H_2_O and Co(NO_3_)·6H_2_O as precursors.^10,29^ For the synthesis of aCNTs about 0.85 g of the Fe–Co/CaCO_3_ bimetallic catalyst powder in a quartz boat, was inserted into the quartz tube and the quartz tube placed into the furnace. The quartz tube was purged with N_2_ at a flow rate of 240 mL min^−1^ while the temperature of the furnace was being ramped up at 10 °C min^−1^ to 800 °C. C_2_H_2_ was then passed through the tube at 100 mL min^−1^ together with N_2_ kept at 240 mL min^−1^ for 2 h before cooling the furnace to room temperature in an inert atmosphere. A similar procedure was followed for the synthesis of aNCNTs except that vapor made from 14.5 M NH_4_OH was passed into the quartz tube with the C_2_H_2_ and N_2_. The aCNTs and aNCNTs were sonicated in aqua regia at 80 °C for 5 h and then rinsed with DI until the pH was neutral. The resultant aCNTs and aNCNTs were dried in an electric furnace at 70 °C overnight.

### 2.3. Synthesis of TiO_2_–aCNT, TiO_2_–aNCNT and NTiO_2_–aNCNT nanohybrids

The sequence in the addition of the reagents up to aging through stirring was adapted from our previous work due to the degree of control on particle size distribution that the method showed.^22^ A weighed amount of aNCNTs was sonicated in 50 mL ethanol (Et·OH) at 40 °C for 1 h while keeping the beaker covered with parafilm to reduce evaporation of the ethanol. The amount of aNCNTs and aCNTs was calculated based on the stoichiometric mass of Ti in the 10 mL titanium butoxide (TiOBut) and was determined to be of a mass equal to 5 wt% of the Ti. A solution of TiOBut (Solution A) was prepared by dissolving 10 mL of TiOBut in a mixture consisting of 100 mL butanol and 10 mL acetylacetone. Another solution (Solution B) of 97% NH_4_OH dissolved in propanol : DI mixture of ratio 1 : 1 was prepared and the Solutions A and B were simultaneously added dropwise into the suspension of aNCNTs under continuous sonication and stirring to form a gray precursor gel. The precursor gel was then removed from the ultrasonicator but stirring continued on a magnetic stirrer for 5 h. The precursor gel was then transferred into silicon carbide-coated Teflon reactor vessels, clamped into a blast-safety casing, and subjected to 30 min of microwave heating at 180 °C and 1 MPa using an MDS-6G synthesis/digestion microwave reactor. The resultant gray precipitate was sequentially washed in propanol, ethanol followed by a final rinse in a 1 : 1 DI : Et·OH solution. After vacuum drying, the precipitate was further dried in an oven at 100 °C for 48 h and crushed using a mortar and pestle. TiO_2_–aNCNT and TiO_2_–aCNT nanohybrids with 5 wt% CNT wt loading were synthesized in a similar way except that TiOBut was hydrolyzed using propanol : DI (1 : 1 v/v) instead of NH_4_OH (Table 1).

**Table 1:** A description of the composition of the powdered nanohybrids and their coloration

Nanohybrid	Description	Color
TiO_2_–aCNT	Titania–amorphous carbon nanotubes	Light gray
TiO_2_–aNCNT	Titania–amorphous nitrogen doped carbon nanotubes	Light gray
NTiO_2_–aNCNT	Nitrogen doped titania–amorphous nitrogen doped carbon nanotubes	Yellow gray

### 2.4. Characterization of nanohybrids

Fourier transform infrared (FTIR) spectroscopic analysis (PerkinElmer Frontier FTIR spectrometer) was carried out to investigate the presence of surface functional groups on the nanohybrids. For transmittance measurements, each sample was ground with KBr and pressed to be a semi-transparent pellet and scanned in the range 450–4000 cm^−1^. Elemental analysis was carried out using an Axs TM® X-ray photoelectron spectrophotometer. Each powdered sample was pressed into a pellet before analysis. Particle size measurements and morphology investigations were carried out using a Jeol TEM 2010 (200 kV). Approximately 5 mg of nanohybrid powder was dispersed in 10 mL ethanol by ultrasonicating at room temperature. Two drops of the colloidal suspension were spread onto a Cu grid that had been previously sputter coated with ∼5 nm carbon. To determine the crystalline phase and to investigate the bonding configuration between NTiO_2_ and aCNTs/aNCNTs, Raman spectroscopic analysis was carried out using an Alpha 300RA AFM/Raman combined system (Witec). A double-sided, transparent tape was spread onto a microscope glass slide and *ca.* 5 mg of the nanohybrid powder was pasted onto the tape. Measurements were taken using the Raman 532 module at an integration time of 0.6 s.

Thermogravimetric analysis (TGA) and differential thermal analysis (DTA) were carried out using a Trios TGA 5500 equipped with a high temperature furnace. For these analyses, *ca.* 10 mg of each nanohybrid was spread onto a platinum high temperature pan before inserting into the high temperature furnace. A ramp rate of 10 °C min^−1^ under a 25 mL min^−1^ N_2_ and air flow were used separately to determine the thermal stability profile in the temperature range 30–850 °C. The TGA and DTA data were analyzed using the Trios TGA analyzer software.

UV-vis absorbance was determined using a PerkinElmer Lambda 650 S UV-vis spectrometer equipped with a tungsten and deuterium lamp with a combined scan range of 180–800 nm. All the scans were done at a rate of 266 nm min^−1^ and all samples were scanned as powders. Photoluminescence spectroscopy was carried out using a Horiba Fluorolog FL3 spectrophotometer equipped with a xenon lamp with scan range of 200–750 nm. The nanohybrids were excited at 350 nm and the emission spectra were collected in the range 270–600 nm. All data, unless specified, was plotted and analyzed using Origin 8.5 Pro. Version.

### 2.5. Photocatalytic performance evaluation

The photocatalytic performance of the nanohybrids was first evaluated for the removal of RR 120 by dispersing 5 mg of each nanohybrid photocatalyst in 200 mL of a 20 ppm solution of the dye. The suspension was sonicated for 30 min at room temperature to achieve adsorption–desorption equilibrium between the dye and the nanohybrid photocatalysts. In the analysis of the efficiency of the nanohybrids, dye removal efficiency was considered during the adsorption–desorption stage and at 30 min after photocatalytic degradation and at the end of contact time *i.e.* 300 min. The nanohybrids were further evaluated for the removal of color from vat-dye laden industrial textile effluent under the similar conditions as in the case of RR 120.

## 3. Results and discussion

### 3.1. Chemical composition analysis of the titania–amorphous carbon nanotube nanohybrids

#### 3.1.1 Analysis of nanohybrids by surface functional groups composition

**Fig. 1:**
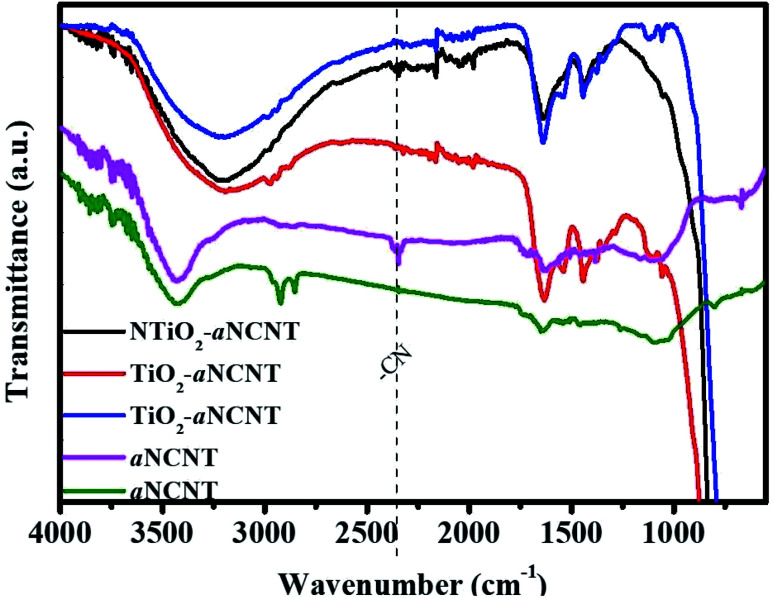
FTIR spectra of the titania–amorphous carbon nanotubes nanohybrids^1^ and amorphous nitrogen doped carbon nanotubes.

FTIR spectroscopic analysis revealed that the aCNTs and aNCNTs showed free and intermolecular bonded alcohol –OH group with peak center at 3450 cm^−1^. However, the nanohybrids have a broad and strong alcohol and carboxylic acid –OH stretch peak in the range 3600–2600 cm^−1^ and a corresponding –OH bending peak at 1637 cm^−1^ (Fig. 1). The FTIR spectra of the titania–amorphous carbon nanotube nanohybrids further confirmed the presence of graphitic forms of carbon residue because of the presence of the alkane C–H stretch peaks at 2980, 2930 and 2879 cm^−1^. In addition, a broad peak in the range 1739–1576 cm^−1^ occurs in all the nanohybrids and the aNCNTs; a region indexed to –C=C–, alkane –C=O and amine –N–H bending. There also occurs a primary alcohol –C–O stretching peak at 1059 cm^−1^ for all the nanohybrids and the aNCNTs. It is also expected that the molecular residue from the alcohol and acetylacetone used during the synthesis process also leave traces of these organic groups on the surfaces of the nanohybrids.

FTIR peaks due to CO and COO^−^ are observed for all nanohybrids at 1115 and 1059 cm^−1^ suggesting that the nanohybrids are capable of adsorbing organic molecules. The peak at 2348 cm^−1^ is assigned to –C–N– and gaseous CO_2_ bonds and it occurs in the aNCNTs and NTiO_2_–aNCNT nanohybrids.^21^ This confirms the doping of N into amorphous C and that the N plays a role in the formation of the TiO_2_/aNCNT interface.

#### 3.1.2 Elemental composition, oxidation state and bonding configuration analysis of the TiO_2_–aNCNT nanohybrids

**Fig. 2:**
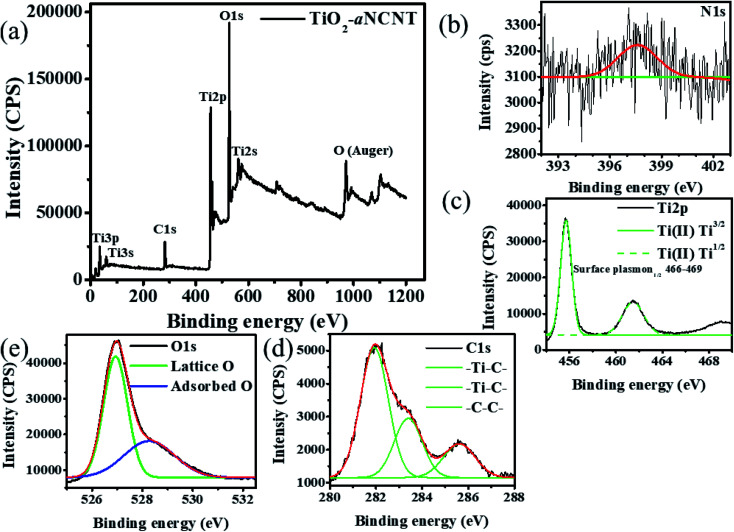
XPS analysis for the nanohybrid TiO_2_–aNCNT showing (a) wide scan, (b) N1s, (c) Ti2p, (d) C1s and (e) O1s spectra.

The wide scan spectrum of the TiO_2_–aNCNT nanohybrids confirmed the exclusive presence of the Ti, O, C and N elements in the nanohybrid at 19.96, 53.87, 25.23 and 0.94 at%, respectively (Fig. 2(a) and Table 2). The N_1s_ spectrum confirmed the incorporation of N into aNCNTs with the occurrence of a broad peak in the range 395–399 eV and centered at 397 eV (Fig. 2(b)). The peak range is indexed to sp^2^ hybridized N bonded to sp^3^ hybridized C, *i.e.* a typical case of N at the periphery of graphitic sheets. The same range is also indexed to binding energy of the –Ti–N– bond^30^ hence suggesting that some of the N atoms on the aNCNT residue are the centers on which TiO_2_ nanocrystals were built. The range is also indexed to C=N which could suggest surface N groups.^26^ The Ti2p spectra has the Ti2p_3/2_ peak center at 455.75 and its Ti2p_1/2_ at 461.47 eV indicating a peak separation of the ∼6 eV and hence indexed to Ti^4+^ (Fig. 2(c)). These peaks indicate a blue shift of the Ti2p_3/2_ and Ti2p_1/2_ in Ti^2+^ which typically occur at 455.03 and 460.85 eV respectively.^31^ A blue shift in binding energies suggests that the elemental species is bonded to a more electronegative element; of which, in the case of TiO_2_–aNCNT nanohybrids, C is the most electronegative on the Pauling scale.^32^ In order of decreasing electronegativity, the elements in the nanohybrid are O (3.44) > N (3.04) > C (2.55) > Ti (1.54). Therefore, the blue shift of the binding energies for Ti2p_3/2_ and Ti2p_1/2_ in Ti^2+^ suggest the occurrence of the Ti–C dative bond.

**Table 2:** Atomic% and elemental mass% concentration of the TiO_2_–aNCNT nanohybrids

	Atomic [%]	Error [%]	Elemental mass [%]	Error [%]
O1s	53.87	0.96	40.39	0.54
C1s	25.23	1.20	14.20	0.77
N1s	0.94	0.46	0.62	0.30
Ti2p	19.96	0.40	44.79	0.57

The occurrence of the –Ti–C– bond is further confirmed by the C1s peaks at 281.92 and 284.41 eV which are indexed to the –Ti–C– bond (Fig. 2(d)). The peak at 283.41 eV has also been found in nanohybrids of TiO_2_–amorphous carbon and has been attributed to the –Ti–C– bond in the configuration –O–Ti–C–.^33^ The C1s spectrum also has a peak in the binding energy range 284.01–286.80 eV, a range in which the binding energy for C in the bonding configurations –C–OH, –C–O–C–, –C–C–, –C–C–H and –C–N falls.^34,35^ The O1s spectrum shows that the nanohybrid consists of both lattice and surface oxygen of which the lattice oxygen is all the oxygen that is found in the TiO_2_ crystalline matrix (Fig. 2(e)). The surface O accounts for 38.7% of all the oxygen and is typically indexed to the functional –OH and –COOH groups. These groups are negatively charged, hence increasing the affinity of the nanohybrids for positively charged species in adoption and photocatalytic processes.

### 3.2. Particle morphology analysis of the titania–amorphous carbon nanotube nanohybrids

**Fig. 3:**
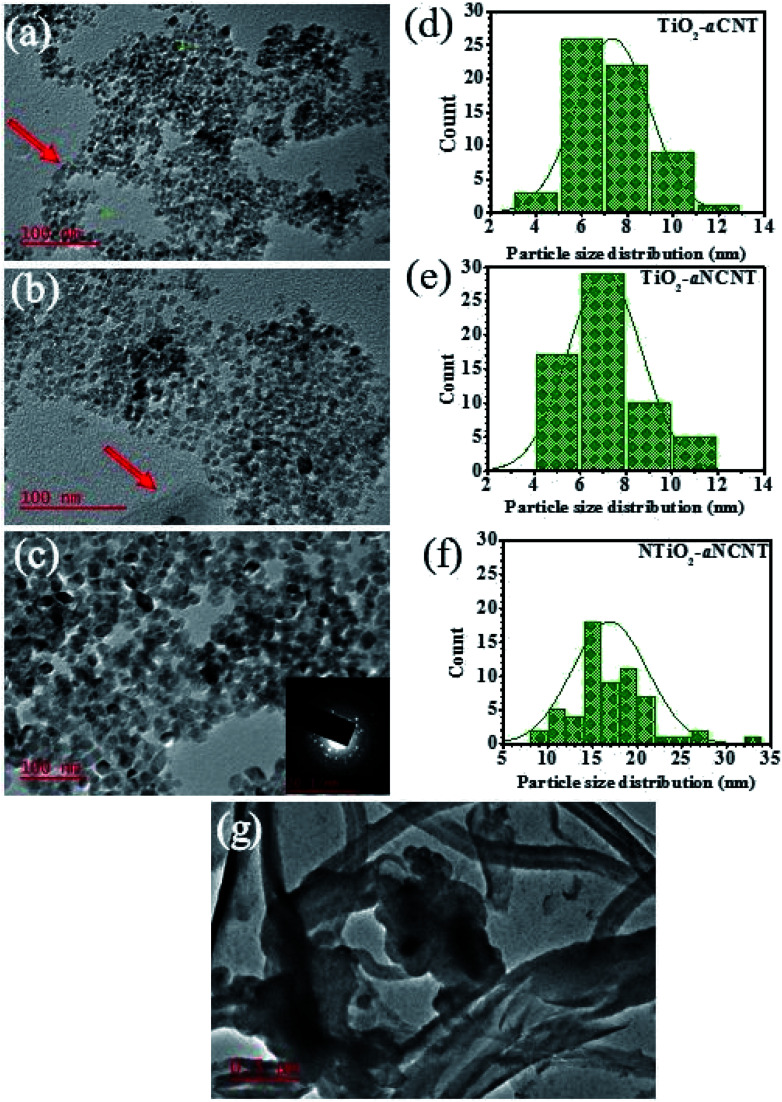
Particle morphology analysis of nanohybrids showing; TEM micrographs for (a) TiO_2_–aCNT, (b) TiO_2_–aNCNT and (c) NTiO_2_–aNCNT nanohybrids; corresponding particle size distribution analysis for each of the nanohybrids (e and f) and (g) TEM micrographs for amorphous carbon nanotubes (aCNTs).

An analysis of the TEM micrographs shows that all the nanohybrids have relatively good particle dispersion hence demonstrating that the synthesis method resulted is well dispersed TiO_2_ nanoparticles. The role of doping either TiO_2_ (to form NTiO_2_) and aCNTs (to form aNCNTs) is revealed in the morphology, particle size and particle size variation of the of TiO_2_/NTiO_2_ in the nanohybrids. TiO_2_ nanoparticles are generally quasi spherical and have small particles with small differences in average particle size and particle size variation (Fig. 3(a) and (b)). The average particle size for the nanohybrid TiO_2_–aCNT is 7.35 ± 1.59 nm and that of the TiO_2_–aNCNT nanohybrids is 7.08 ± 1.60 nm (Fig. 3(d)). The NTiO_2_–aNCNT nanohybrids, on the other hand, has larger NTiO_2_ nanoparticles (16.90 ± 4.11 nm) with various shapes including cuboidal, rhomboid, quasi spherical and rod-shaped and hence a wide particle size distribution Fig. 3(c). Unlike the other nanohybrids, the NTiO_2_ nanoparticles all have defined crystal shapes with distinct edges. These features for NTiO_2_ are consistent with our previous observations and work by other researchers.^36,37^ As such, it can be established that the hydrothermal synthesis of N doped TiO_2_ leads to the formation of cuboidal NTiO_2_ nanoparticles. The small size of the TiO_2_–aCNT and TiO_2_–aNCNT nanohybrids make them better suited for applications where adsorption is a key step since smaller sized nanoparticles offer a higher surface area for adsorption.^38^

**Fig. 4:**
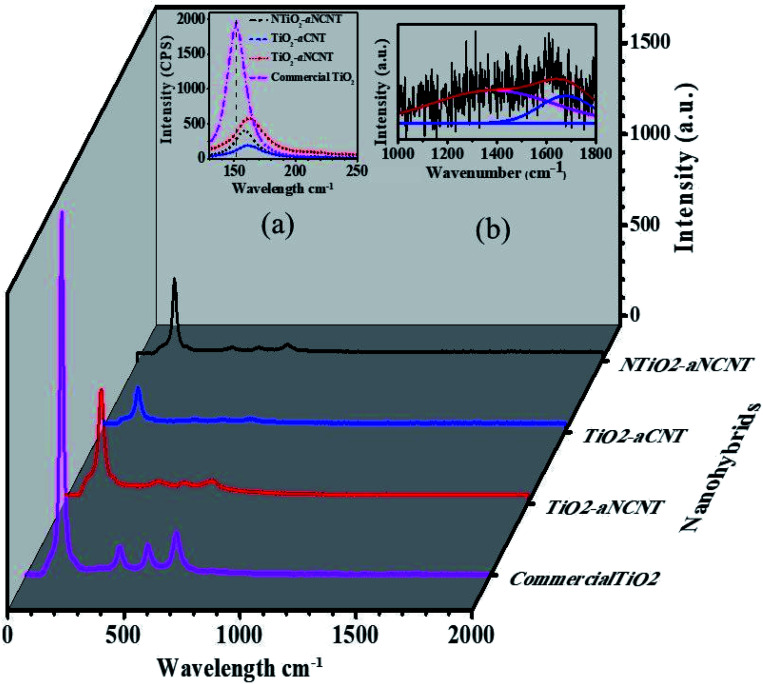
Raman spectra for the nanohybrids and commercial TiO_2_ with insets showing (a) the shifts in wavenumber due to hybridization (b) D- and G-bands for NTiO_2_–N-CNTs after functionalization.

The TiO_2_–aCNT and TiO_2_–aNCNT nanohybrids show traces of aCNTs and aNCNTs, respectively (indicated by red arrows on the (Fig. 3(a) and (b)). In the former, the aCNT is densely covered by TiO_2_ nanoparticles resulting in the general tubular alignment of the TiO_2_ nanoparticle clusters whereas in the latter, the aNCNT is sparsely covered. The nanohybrid NTiO_2_–aNCNT, on the other hand, shows no traces of aNCNT structures although alkane and alkene peaks attributed to graphitic sheets and CNTs are observed in the FTIR spectra of the nanohybrid (Fig. 1). These observations are consistent with the Raman spectra of the nanohybrids, where D-and G-bands could be deconvoluted in the TiO_2_ based nanohybrids but not in NTiO_2_–aNCNT (Fig. 4 inset (b)). The absence of the aCNTs and the aNCNTs is due to their degradation, which results in their graphitic and amorphous residue being incorporated into the TiO_2_/NTiO_2_ nanocrystals. Lastly, selected area diffraction of the NTiO_2_ nanoparticles agrees with the Raman peak intensities in indicating good crystallinity of the nanohybrids (Fig. 3(c) inset) and Fig. 4). Therefore, the microwave assisted hydrothermal synthesis method is shown to be efficient in tailoring of doped and undoped TiO_2_–aCNT/aNCNT nanohybrids.

### 3.3. Raman spectroscopy analysis of nanohybrids

All the nanohybrids are of the anatase phase as indicated by the anatase E_g_, A_1g_ and B_1g_ peaks arising from the symmetric stretch, antisymmetric bending and symmetric bending of the –O–Ti–O– bond respectively.^39^ The weak intensity of the E_g_ peaks indicate that the hydrothermally synthesized nanohybrids have lower crystallinity than commercial anatase TiO_2_. All the nanohybrids show a red shift of the E_g_ anatase peak compared to the E_g_ peak of commercial anatase TiO_2_. The anatase E_g_ peak for commercial TiO_2_ appears at 152 cm^−1^ while the nanohybrids appear at higher wavelengths in the decreasing order TiO_2_–aNCNT (163 cm^−1^) > TiO_2_–aCNT (160 cm^−1^) > NTiO_2_–aNCNT (157 cm^−1^) (Fig. 4 inset (a)). The degree of the shift of the wavenumber denotes the degree to which the electronic structure of TiO_2_ has been altered and thus, in the case of the nanohybrids, the strength of the bonds between TiO_2_/NTiO_2_ nanoparticles and the aCNTs/aNCNTs.^33,34^ Therefore, results indicate that the bond strength is in the decreasing order TiO_2_–aNCNT (163 cm^−1^) > TiO_2_–aCNT (160 cm^−1^) > NTiO_2_–aNCNT (157 cm^−1^); making the aNCNT to be the most suitable for forming a more stable TiO_2_–CNT interface. The stronger bonding between aNCNTs and TiO_2_ can be attributed to the introduction of partial positive charges (*δ*^+^) over C atoms and partial negative charges (*δ*^−^) over N atoms in the N-CNT skeleton. These charges reduce the surface energy of the NCNTs compared to CNT and thus making it energy favorable for the dative covalent attachment of TiO_2_ and NTiO_2_ seed crystals during the initial polymeric condensation of NTiO_2_ onto the CNTs.

The D- and G-bands of the nanohybrids were of low intensity due to the disintegration of the carbon nanotubes and its integration into the TiO_2_/NTiO_2_ crystals and hence curve fitting was done to determine their exact peak position and intensities (Fig. 4 inset (b)). The disappearance of the D- and G-bands due to aCNTs is in agreement with previous observations where MnO was embedded onto aCNTs.^40^ Curve fitting of the Raman spectra of the nanohybrids showed weak D- and G-peaks for TiO_2_–aCNT and TiO_2_–aNCNT nanohybrids such that analysis of these peaks was not possible. An analysis of peak intensities and D- and G-band band positions was carried out to determine the degree of bonding between TiO_2_/NTiO_2_ nanoparticles with aCNTs and the aNCNTs. Previous studies have shown that the modification of CNTs with heteroatoms and heteroatomic groups alters the electronic properties of the CNTs thus interfering with both the position and intensity of the graphitic D- and G-bands.^7,41,42^ An attachment of electron acceptor groups results in a blue shift of the D- and G-bands while electron donor groups produce a red shift.^41,42^ The D- and G-bands of the nanohybrid NTiO_2_/TiO_2_–aCNT/aNCNT had red shift of both the D- and G-bands relative to those of aNCNTs from 1397 to 1382 cm^−1^ for the D-band and 1589 to 1676 cm^−1^ for the graphitic band (Table 3). This could suggest that the bonding between NTiO_2_/TiO_2_ and aCNT/aNCNT occurs through the –OH groups on the Ti(OH)_4_ intermediate donating their lone pair of electrons to the surface –OH and –COOH groups of the aNCNTs and aCNTs. For the aNCNTs, the –OH groups on the Ti(OH)_4_ intermediate could also donate their lone pair of electrons to the *δ*^+^ surface C atoms to form a dative covalent bond. This additional bonding configuration is expected to make the bonding between TiO_2_ and aNCNTs to be stronger as suggested by the greater change in the wavenumber for the anatase E_g_ band due to the nanohybrid TiO_2_–aNCNT. This is despite the dative bond between Ti^4+^ and the N-CNTs withdraws electrons from the NCNTs. The extra electrons due to the presence of N in the N-CNTs and the presence of N in NTiO_2_ alters the electronic behavior of the nanohybrid and hence its response to the light, including the exciting laser during Raman spectroscopic analysis. This points to the degree by which doping both TiO_2_ and CNTs alters the optical properties of the nanohybrid.

**Table 3:** An analysis of changes in band position and intensity changes for the D-and G-bands

Nanohybrid	Properties	D-band	G-band
aNCNTs	Wavelength pos. (cm^−1^)	1367	1589
	Band area (a.u.)	4748	1293
	FWHM (cm^−1^)	317	100
	*I*_G_/*I*_D_	0.272	
aCNTs	Wavelength pos. (cm^−1^)	1357	1596
	Band area (a.u.)	9303	4269
	FWHM (cm^−1^)	234.2	96.8
	*I*_G_/*I*_D_	0.458	
NTiO_2_–aNCNT	Wavelength pos. (cm^−1^)	1382	1676
	Band area (a.u.)	392	1092
	*I*_G_/*I*_D_	0.359	
TiO_2_–aCNT	N/A		
NTiO_2_–aCNT	N/A		

### 3.4. Thermogravimetric analysis of nanohybrids

**Fig. 5:**
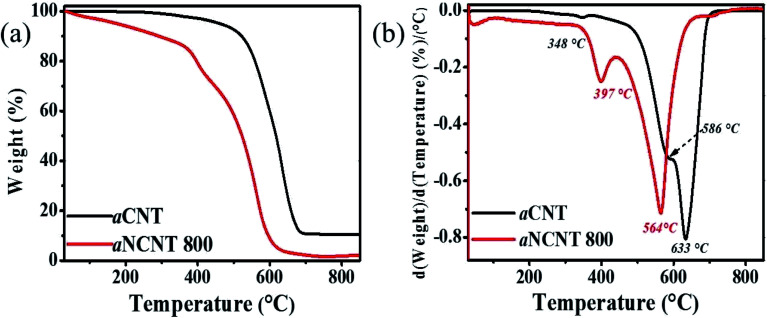
TGA (a) and DTA (b) thermograms for aNCNT and aCNTs.

TGA and DTA thermograms for aCNTs and aNCNT confirm the coexistence of an amorphous carbon and the crystalline forms in each sample. It is further revealed that both amorphous and crystalline forms of aNCNTs degrade at lower temperatures compared to those of aNCNTs. Furthermore, the amorphous nature of aNCNTs relative to aCNTs is demonstrated by a lower intensity of the differential peak corresponding to crystalline aNCNTs being lower that corresponding to crystalline aCNTs (Fig. 5(b)).

**Fig. 6:**
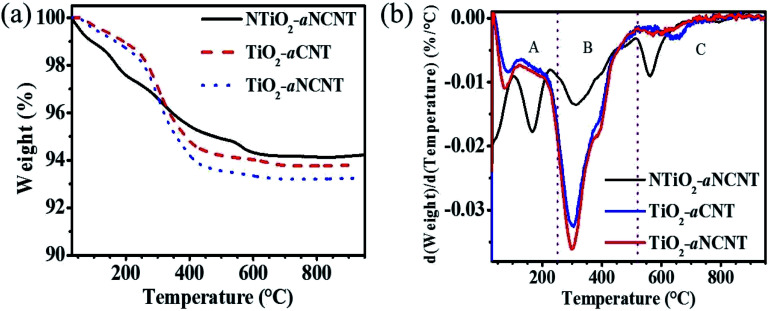
A comparison of TGA (a) and DTA (b) profiles for nanohybrids.

DTA and TGA thermograms of the nanohybrids show that all the nanohybrids have a dual composition of amorphous carbon and crystalline CNTs and NCNT, with the latter having the highest wt% composition in each hybrid (Fig. 6(a) and (b)). The increase in the DTA peak due to amorphous residue from both the aNCNTs and aCNTs relative to the DTA peak corresponding to the crystalline forms suggest that the nanohybrid synthesis process resulted in the breaking down of part of the crystalline forms into amorphous carbon residue and hence the aim of this work achieved. Previous reports have demonstrated that CNTs and aNCNTs synthesized at high temperatures, at long reaction times, high flow rates of the carbon source are prone to having thick flaky walls whose outer wall is weakly held to the inner crystalline form.^10^ The nanohybrids also demonstrate high adsorption for water and polar organics because of the wt% loss in the temp range 30–250 °C (Table 4 and Fig. 6b). This suggest that they are suited for applications where adsorption is part of the mechanism *e.g.* photocatalytic applications and sensing with NTiO_2_–aNCNT showing a greater adsorptive capability. The occurrence of these surface groups is also corroborated by the broad –OH vibration peak in all the nanohybrids in the FTIR spectra for the nanohybrids (Fig. 1). For the nanohybrid TiO_2_–aNCNT the occurrence of the surface groups is further confirmed by the occurrence of adsorbed oxygen peaks in the O1s spectra (Fig. 2(f)). The degradation temperature peaks for the crystalline forms of the aNCNTs and aCNTs occur at slightly lower temperatures in the nanohybrids compared to the aNCNTs and aCNTs. This is attributed to the TiO_2_ and NTiO_2_ trapping more heat at any given temperature and hence delivering more heat to the carbonaceous forms compared to the case where the carbonaceous forms are without TiO_2_.^42^ The amorphous form of aCNTs and aNCNTs are thermally degraded at a lower temperature range in the nanohybrids (224–448 °C) than in the aCNTs (550–650 °C) (Fig. 5(b)). However, these amorphous forms in the nanohybrids degrade in the same temperature for all the nanohybrids and over a wider range. In addition, the DTA thermograms have a shoulder peak occurring at 380 °C. The longer degradation range and the occurrence of the shoulder peak suggests that the two components are being degraded in the temperature range 224–448 °C. We reason that the shoulder peak at 380 °C is due to graphitic flakes peeling off from outer walls of the thick aNCNT and aCNTs during the synthesis of the nanohybrids while the lower temperature range is due to the amorphous form and towards the extreme low of this DTA peak, it is the carbonaceous fragments and atoms intercalated into the TiO_2_ lattice.

**Table 4:** Thermogravimetric analysis of wt% loss for nanohybrids at selected temperature ranges

Nanohybrid	wt% loss per temperature range (%)		
	<250 °C	25 < 520 °C	>520 °C
TiO_2_–aCNT	1.27	4.73	0.377
TiO_2_–aNCNT	1.50	5.03	0.299
NTiO_2_–aNCNT	2.56	2.52	0.681

In retrospect, the DTA and TGA thermograms of the nanohybrids suggest that their chemical and mechanical integrity in air has its upper threshold at 200 °C. At higher temperatures, the doped ∼C and the amorphous carbon residue is oxidized yet these are essential for the maintenance of visible light absorption, and low charge recombination. Also, the changes in the thermal degradation temperatures in the region for degradation for aNCNTs and aCNTs lead to the inference of the strength of the interfacial bond between the TiO_2_ or NTiO_2_ nanoparticles and the aCNTs. The greater the shift from the thermal degradation of the aCNTs, the stronger the interaction between the aCNTs and the TiO_2_ nanoparticles. The idea works on the assumption that the chemical interaction at the interface between the CNTs and the TiO_2_ introduces a new set of bonds with unique bond strengths and enthalpies. Therefore, these deviations of the thermal degradation temperatures suggest that the nanohybrid between NTiO_2_/TiO_2_ and aNCNTs/aCNTs has been formed.^34,42^

### 3.5. Optical response of the nanohybrids

#### 3.5.1 UV-vis spectroscopic analysis

The synergistic effect of doping TiO_2_ with N and making its composite with aNCNT enhanced the energy band-gap reduction better than hybridizing TiO_2_–aCNTs. This is because the nanohybrid NTiO_2_–aNCNT has a lower energy band gap of 2.97 eV while the nanohybrids TiO_2_–aNCNT and TiO_2_–aCNT have equal energy band gaps of 3.00 eV. A reduction in the energy band gap is the main indicator of the absorption edge of semiconductor photocatalysts and a low *E*_g_ indicates a higher absorption edge. In the case of these nanohybrids, the energy band gap for all the nanohybrids indicates that all the nanohybrids absorb in the visible region *i.e.* beyond 390 nm which is the typical band edge for undoped and perfectly crystalline TiO_2_. The band gaps for the nanohybrids NTiO_2_–aNCNT, TiO_2_–aNCNT and TiO_2_–aCNT correspond to the absorption edges 430, 424 and 424 nm, respectively; hence increased absorption of visible light compared to commercial TiO_2_ (with absorption edge at 390 nm).

**Fig. 7:**
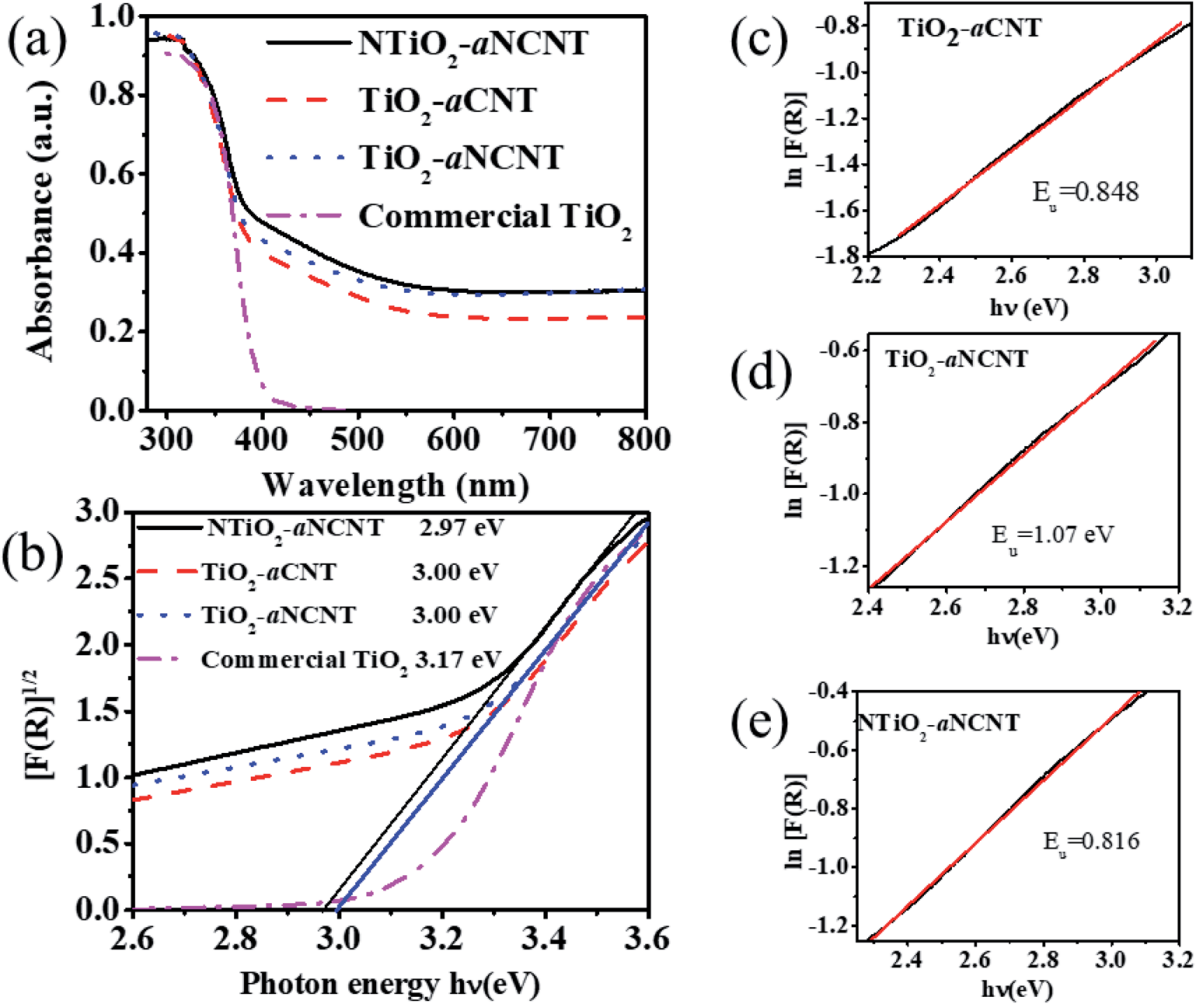
Photoresponse of the nanohybrids as depicted by the (a) UV-vis spectrum and (b) Tauc plots showing examples of how the energy band gap is determined by the tangential lines and (c)–(e) Urbach energy (*E*_u_) plots for the nanohybrids.

Hybridizing TiO_2_ with aNCNTs or aCNTs produced nanohybrids with increased photosensitization in the visible range as shown by an increase in absorbance from 0 to the range 0.23–0.303 for the nanohybrids. aNCNTs have a higher photosensitization effect on TiO_2_ than aCNTs and doping of TiO_2_ with N has insignificant contribution into photosensitization of the nanohybrids as shown by the insignificant difference in the visible light absorbance for the nanohybrids TiO_2_–aNCNT (0.293 a.u.) and NTiO_2_–aNCNT (0.303) (Fig. 7(a)).

Photosensitization of the nanohybrids is further enhanced by the Urbach energies of the nanohybrids. Urbach energy (*E*_u_) is evidenced by the occurrence of Urbach tails at the band edge of semiconductor UV-vis spectra.^37,43^ At constant temperature *E*_u_ is due to crystalline defects. These defects can be due to the transformations in the crystalline structure due to temperature changes, occurrence of dopants (which distort the crystalline structure) and amorphosity of the nanocrystals.^44,45^ In semiconductor photocatalysis, *E*_u_ enhances the absorption of light beyond the absorption edge. *E*_u_ is the reciprocal of the slope in a plot of the natural log of the absorption coefficient [ln(*R*)] and photon energy (*hν*).^45^ The results show that the hybridizing TiO_2_ with aNCNTs is effective in enhancing visible light absorption through synergistic effect of doping TiO_2_ with N and hybridizing it with aNCNTs is effective in enhancing visible light absorption through *E*_u_. This is shown by the nanohybrid TiO_2_–aNCNT having the highest *E*_u_ of 1.07 eV (Fig. 7(d)). However, the synergy of doping TiO_2_ with N and hybridizing with aNCNT suppresses the effect of aNCNTs in increasing the *E*_u_ as shown by the low *E*_u_ for the nanohybrid NTiO_2_–aNCNT (0.816 eV) compared to that of TiO_2_–aCNT (0.848 eV).

#### 3.5.2 Photoluminescence spectrum for nanohybrids

**Fig. 8:**
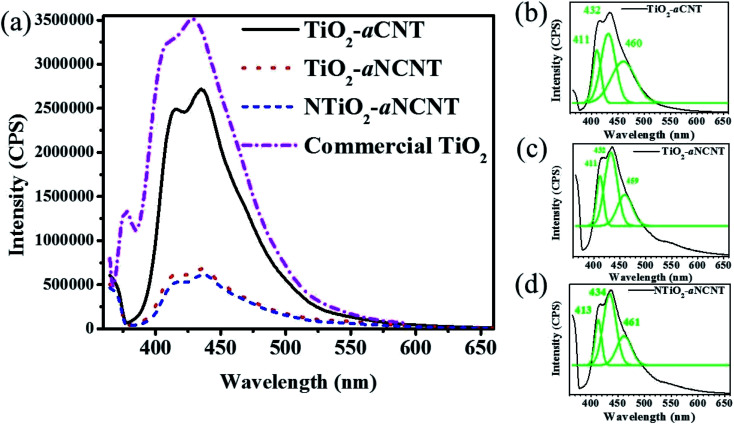
Optical response of the nanohybrids as determined by (a) photoluminescence spectra of all the nanohybrids and deconvoluted spectra of the nanohybrids (b) TiO_2_–aCNT, (c) TiO_2_–aNCNT and (d) NTiO_2_–aNCNT.

The PL intensity indicates that hybridizing TiO_2_ and NTiO_2_ with aNCNT/aCNT suppresses charge recombination compared to commercial TiO_2_ (Fig. 8(a)). However, there is a significant difference in the degree to which aCNT and aNCNT suppress charge recombination in that aNCNT-based nanohybrids (NTiO_2_–aNCNT and TiO_2_–aCNT) have a significantly low PL intensity than the nanohybrid TiO_2_–aCNT whose band edge peak has an intensity of 2.71 × 10^6^ CPS. Furthermore, a positive synergy exists between doping TiO_2_ with N and hybridizing with aNCNT in that the nanohybrid NTiO_2_–aNCNT has the lowest PL intensity of 6.24 × 10^5^ CPS; a significantly low intensity compared to that of TiO_2_–aNCT 6.59 × 10^5^ CPS. The implications of the PL intensity on the photocatalytic activity of the nanohybrids is discussed in the sections for the photocatalytic activity of the nanohybrids (Section 3.6).

The PL spectra of the nanohybrids is also used to infer the presence of crystal defects and uncapped bonds on the surface of crystalline materials such as TiO_2_. Charge recombination occurring at crystal defects is indicated by the peaks occurring at 460 nm while charge recombination due to ∼dangling bonds is indicated by PL spectra not going back to 0 CPS at the longer wavelengths.^46^ Therefore, the PL spectra for the nanohybrids indicates that they have O vacancies but do not have any dangling bonds (Fig. 8(b)–(d)). A controlled occurrence of these O vacancies can enhance the photocatalytic activity of TiO_2_ in that, these sites introduce mid-band gap states closer to the VB hence providing an alternative conduction band in a lower energy state. The overall effect is the reduction of the *E*_g_.^47^ However, as it is in the case of most dopants, this reduction in the *E*_g_ is beneficial if there is an efficient means of sequestering photogenerated electrons away from the VB of the TiO_2_ since there charge recombination is reduced. It is, therefore, expected that the occurrence of these vacancies in the nanohybrids TiO_2_–aNCNT and NTiO_2_–aNCNT enhances the photocatalytic activity due to the low charge recombination.

### 3.6. Photocatalytic performance of the nanohybrids

#### 3.6.1 Removal of the dye Reactive Red 120 (RR 120) from a single component system

##### A. Adsorptive removal of the dye RR 120

**Fig. 9:**
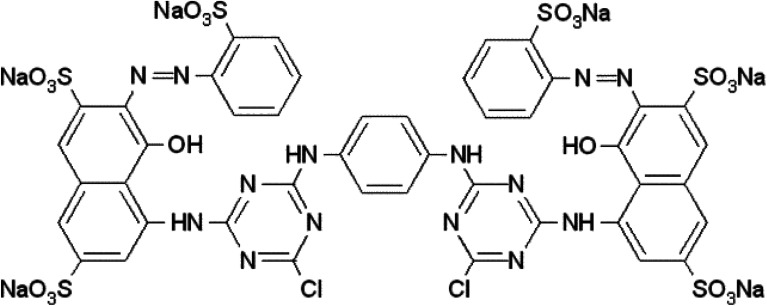
Reactive Red 120 (CI) molecule.

**Fig. 10:**
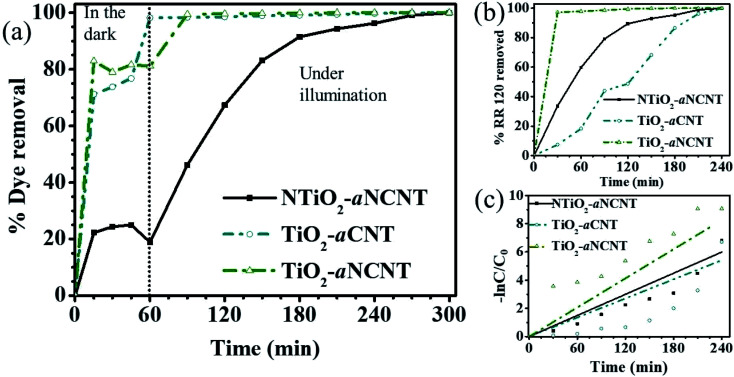
Photocatalytic performance of the nanohybrids measured by (a) % dye removal during adsorption–desorption equilibration (in the dark) and photocatalytic degradation (under illumination), (b) photocatalytic degradation under illumination and (c) apparent first order rate constants (*k*_app_) for photocatalytic degradation under illumination.

The nanohybrid TiO_2_–aCNT had the highest adsorptive dye removal of 98.2% at 60 min of adsorption–desorption equilibrium (Table 5). While the nanohybrid TiO_2_–aNCNT, showed the second highest overall adsorptive removal of the dye (81.1%), it showed the fastest adsorptive removal, reaching 82.9% within 30 min (Fig. 10(a)). The nanohybrid NTiO_2_–NCNT showed the lowest adsorptive removal and the highest leaching of the dye molecules reaching its maximum at 45 min and subsequently leaching off 6% of the already adsorbed dye. Given that adsorption is a factor of active surface area and charge difference between the catalyst and the pollutant, the high adsorptive capacity of the nanohybrids TiO_2_–aCNT and TiO_2_–aNCNT is typical of catalysts with a large active surface area.^48^ The surface is a factor of particle size, particle size distribution and dispersion. Small, monodisperse and well dispersed nanoparticles offer high active surface area. The adsorptive behavior of the nanohybrids behave as predicted by the TEM micrographs of the nanohybrids in that the nanohybrids TiO_2_–aCNT and TiO_2_–aNCNT have the smallest particle size (7.35 ± 1.5 and 7.07 ± 1.6 nm, respectively) and are better dispersed (Table 5 and Fig. 3). NTiO_2_–aNCNT, on the other hand, has the highest particle size distribution (16.9 ± 4.1 nm) and the lowest adsorption. It also suggests that at the operational pH for the adsorption, the TiO_2_–aNCT and TiO_2_–aCNT nanohybrid photocatalysts have an overall negative charge, hence they actively attract the dye molecules. Charge behavior of the dye RR 120 and its interaction with CNT-based photocatalysts has shown that the attractive forces are due to the positively charged sulfite groups (R-SO_3_^−^) after the dissociation of the Na^+^ ion and the – interactions arising from π–π the cyclic rings in the RR 120 molecule (Fig. 9).^49^ Also, being an azo dye, the RR 120 molecule is rich in N and as such it is expected that in its interaction with the N-doped photocatalysts, the lone pairs of the N atoms will result in the repulsion between the dye molecules and the photocatalysts. The low adsorptive capacity of NTiO_2_–aNCNT and the highest adsorption of TiO_2_–aCNT could suggest that the repulsion due to these lone pairs is more pronounced than the possible attractive forces due to the – interactions between the cyclic carbon rings in the dye π–π molecules and the –C–C– making up of the aNCNTs and aNCNTs.

**Table 5:** A summary of the optical properties and average particle size compared to the adsorptive and photocatalytic performances of the nanohybrids

Nanohybrid	*E*_g_ (eV)	PL intensity (×10^−6^ CPS)	*k*_app_ (×10^−2^ min^−1^)	Particle size (nm)	% dye adsorption	% dye removed at 30 min under illumination
TiO_2_–aCNT	3.00	0.272	2.25	7.35 ± 1.59	98.2 ± 2.0	99 ± 1.75
TiO_2_–aNCNT	3.00	6.8	3.44	7.07 ± 1.60	81.1 ± 1.55	99 ± 1.65
NTiO_2_–aNCNT	2.97	6.07	2.49	16.9 ± 4.1	18.9 ± 1.91	46 ± 2.3

##### B. Photocatalytic removal of the dye RR 120 under illumination

The TiO_2_–aNCNT nanohybrids demonstrates the highest photocatalytic degradation of the dye jumping from 81.1% to 99.1% after 30 min under illumination while the removal by TiO_2_–aCNT only reaches 99% dye removal after 120 min of illumination (Fig. 10(b), (c) and Table 5). The nanohybrid NTiO_2_–aNCNT shows improved photocatalytic removal by reaching 99.9 overall dye removal at contact time 300 min like all the nanohybrids at an overall apparent first order constant of 2.49 × 10^−2^ min^−1^, a rate constant that is higher than that for TiO_2_–aCNT (2.25 × 10^−2^). Kinetic studies of the photocatalytic removal of the dye demonstrate that the photocatalytic degradation of the dye follows pseudo first order kinetics.

A comparison of the photocatalytic performance of these nanohybrids with the physicochemical properties of the nanohybrids leads to the following observations:

(i) The dye removal performance for the TiO_2_–aCNT nanohybrids is more dependent on the small particle size, monodispersity and good particle dispersion (properties which insinuate a large surface area) more than it does rely on the optical properties;

(ii) The NTiO_2_–aNCNT nanohybrids, however, relies more on its good photocatalytic properties such as low *E*_g_ and high photosensitization in the visible to near infrared region. These properties make it more suited for visible light photocatalysis than all the nanohybrids. This means that the photocatalytic degradation by the TiO_2_–aNCNT photocatalytic nanohybrids proceeds through the production of the reactive oxidation species (O_2_˙^−^ and ^−^˙OH) more than the surface mediated redox degradation of the nanohybrids;

†The spectra of the samples are named according to the stage and time at which each sample was collected *i.e.* adsorptive (*A*) for adsorption–desorption equilibration and photocatalytic (*P*) for degradation under illumination. The times at which the samples are collected at each stage are indicated in Arabic numerals.(iii) The TiO_2_–aNCNT nanohybrids have high visible light photosensitization, high *E*_u_ but high *E*_g_; characteristics which make it relatively better suited for visible light photocatalysis than TiO_2_–aCNT and NTiO_2_–aNCNT photocatalytic nanohybrids. The nanohybrid TiO_2_–aNCNT also has the smallest particle size and good particle size distribution. Therefore, it is the most suited nanohybrid for photocatalytic degradation because it possesses both the good characteristics and not the downside characteristics from the latter.

#### 3.6.2 Photocatalytic treatment of industrial textile effluent waste

**Fig. 11:**
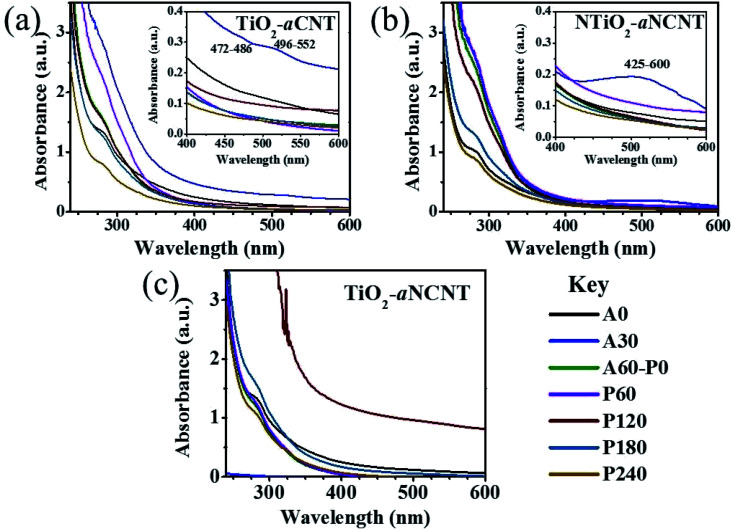
UV-vis spectra† of depicting degradation profiles for textile wastewater during photocatalytic treatment of the (a) TiO_2_–aNCNT (b) NTiO_2_–aCNT and (c) TiO_2_–aNCNT nanohybrids. Insets show the formation of peaks in the process of the oxidative treatment.

The UV-vis spectra for the textile wastewater suggest that the water consists of mildly coloring dissolved organics. The color is assumed to be due to the presence of VAT dye nanoparticles. Typical textile wastewater also consists of organics such as aldehydes, alcohols, ketones and a wide range of organics from surfactants.^50,51^ Permissions to get the exact constituents of the dyeing, washing, rinsing and scouring chemicals could not be obtained from the source textile factory. The UV-vis spectra for the degradation of the organics in the wastewater suggest that it proceeds through the formation of intermediates that either absorb at a different wavelength or react with each other to form a secondary intermediate that absorbs at a different wavelength. This is indicated by the occurrence of isosbestic points between the spectra of aliquots collected at different times for each of the nanohybrids. Isosbestic points are an indication of the changing composition of a sample.^52^ The quantity of some of these intermediates is prominent such that new peaks in the visible range appear for the TiO_2_–aCNT and NTiO_2_–aNCNT nanohybrids in the ranges 472–486 and 425–600 nm, respectively, after 30 min during the adsorption–desorption equilibrium attainment stage (A30) (Fig. 11(a) and (b)). These peaks suggest that there some of the polluting components in the wastewater are broken down even in the dark by these nanohybrids. A classic example of a mass transfer observable in the UV-vis spectrum in the degradation of N-containing groups such as those typically contained by surfactants, to form nitrile and nitrate ions.^53^ Other C-containing moieties found in surfactants also form-visible light-absorbing groups.^52^

Another feature common to all the TiO_2_–CNT nanohybrids is the fluctuation of the absorbance spectra for aliquots. For example, the aliquot collected at 30 min during sonication in the dark for the TiO_2_–aCNT nanohybrid has a high absorbance than the wastewater before degradation (Fig. 11).

Similar observations are made on the nanohybrid NTiO_2_–aNCNT. The nanohybrid TiO_2_–aNCNT shows the highest adsorptive removal of the organics from the wastewater within the first 30 min. This is evidenced by the decrease of the absorbance to almost zero across the whole spectrum Fig. 11(c). In addition to this, the aliquot collected after 120 min of degradation under illumination (P120) has the highest absorbance (Fig. 11(c)). These features also point to the possibility of the formation of UV-absorbing intermediates through a chemical reaction of intermediates formed from the degradation of non-UV absorbing organic moieties.^54^ VAT dyes, which are insoluble, can be broken down to water-soluble intermediates through the action of the radicals produced in AOPs. This would result in an increase in the concentration of dissolved organic moieties and hence an increase in the overall absorbance spectrum.^55^ However, in all the nanohybrids, the final aliquots collected at 240 min of degradation under illumination (P240) has the lowest absorbance. This points to the fact that the concentration of the organic pollutants in the wastewater has decreased and hence in overall the catalysts are showing catalytic degradation for the organics in the textile wastewater. In essence, the nanohybrids are shown to effectively break down textile dyes in industrial wastewater. High photocatalytic and adsorptive activity is desirable for photocatalysts to be incorporated into photocatalytic reactor membranes in order to accommodate the typical reduction in photocatalytic activity of the photocatalyst once in the PMR as it has been observed before.^56^ The proposed photocatalysts have the additional advantage of demonstrating the catalytic degradation of VAT dyes in industrial textile water as suggested by the formation of visible light absorbing intermediates. In as much different photocatalysts are generally evaluated for efficacy under different environments, the photocatalytic activity of TiO_2_–aCNT and TiO_2_–aNCNT nanohybrids on single dye systems is comparable to the activity of photocatalytic nanohybrids that are made from crystalline CNTs (Table 6). We attribute the good activity to the high adsorption of the dye by these catalysts; a feature that is unique to these photocatalytic nanohybrids. This makes them offer a niche for the use of photocatalytic membrane reactors that can be used to form potentially useful intermediates.

**Table 6:** A comparison of the efficiency of the current work to previous literature

Photocatalyst	Application	Result	Ref.
Magnetic MWCNT–TiO_2_	Photocatalytic degradation of malachite green (MG)	100% 20 ppm removal of MG at pH5 and catalyst loading of 200 ppm catalyst loading	57
		Catalyst reusable despite the bulk of TiO_2_–MWCNT and simple electrostatic attraction	
TiO_2_–Pt–MWCNT	Photocatalytic hydrogen production under irradiation	2327 and 2091 μmol g L^−1^ H_2_ produced in 2 h under irradiation of glycerol and methanol respectively	58
Ag_3_PO_4_@MWCNTs@Cr:SrTiO_3_	Photocatalytic degradation of malachite green	100% removal of MG in 6 min at 100 mg pollutant per 1 g of catalyst under solar irradiation	59
Ag_3_PO_4_@NC	Photocatalytic degradation of norfloxacin, diclofenac and phenol	*k*_1app_ = 1.248, 0.925, and 0.721 min^−1^ for the degradation of, norfloxacin, diclofenac and phenol	26
		All Ag_3_PO_4_@NC nanohybrids performed better than Ag_3_PO_4_	
Amorphous C doped Zn_*x*_Cd_1−*x*_S	H_2_ evolution under visible light	Energy band gap reduction from 2.7 to 2.3 eV and up to 280% increase in H_2_ evolution with incorporation of amorphous Carbon	60
Sn@aCNT	Lithium-ion storage batteries	Specific capacity of 749 mA h g^−1^ at current density of 0.2 A g^−1^	11
Amorphous carbon doped ZnO/Zn	Photocatalytic degradation of basic blue 41 (12.5 ppm) and 100 mg L^−1^ catalyst loading under visible light	7.046 × 10^−1^ ppm dye adsorption in 60 min and 90% dye removal in 180 min at *k*_1app_ = 17.2 × 10^−3^ min^−1^	61
TiO_2_–aCNT, TiO_2_–aNCNT, NTiO_2_–aNCNT (radiation microwave hydrothermal synthesis)	Photocatalytic degradation of Congo red dye under LED white light	Dye removal efficiency at 30 min adsorption and 120 min photodegradation TiO_2_–aCNT – 69.4 and 92.5%, TiO_2_–aNCNT – 89.2 and 99.2%, NTiO_2_–aNCNT – 42.9% and 82.6%	22
TiO_2_–aCNT, TiO_2_–aNCNT, NTiO_2_–aNCNT	Photocatalytic degradation of RR 120 (20 ppm) at 100 ppm catalyst loading	Dye removal efficiency at 60 min adsorption and 300 min photodegradation TiO_2_–aCNT – 98.2 and 99.9%, TiO_2_–aNCNT – 81.1 and 99.9% NTiO_2_–aNCNT – 18.9% and 99.9%	Current work

## 4. Conclusions

This work has demonstrated that amorphous CNTs, which have been previously deemed to be of less value, can tailor the optical properties of TiO_2_ to enable visible light absorption through energy band gap reduction and visible light sensitization. In addition to this, these nanohybrids were synthesized through a simple and more reproducible microwave assisted hydrothermal method. The nanohybrids with the smallest particle size, narrowest particle size distribution, highest visible light sensitization, and lowest energy band gap were found to have the highest photocatalytic activity. The nanohybrids with a small particle size, good particle size dispersion but lower ability to absorb visible light have the highest adsorptive capacity and lowest degradation rate constant. Finally, these nanohybrids show a degree of photocatalytic activity for the degradation of organic pollutants in industrial wastewater. Herein, we present another step in the direction towards energy efficiency, and economic industrial wastewater treatment through photocatalysis. The small particle size, narrow particle size distribution and the intimate contact between TiO_2_ and the graphitic material from aCNTs makes the subject nanohybrids better suited for the fabrication of photocatalytic reactor membranes with fewer micropores at the TiO_2_ polymer interface. The formation of visible light absorbing intermediates also opens a niche for the use of these nanohybrids in the fabrication of photocatalytic membrane reactors that can drive reactions for the generation of potentially useful intermediates from wastewater.

## Conflicts of interest

There are no conflicts to declare.

## Supplementary Material
